# “IBASHO” AND THE OBJECTIVE WELL-BEING OF OLDER ADULTS IN A MARGINALIZED COMMUNITY

**DOI:** 10.1093/geroni/igac059.3001

**Published:** 2022-12-20

**Authors:** Yuichi Watanabe

**Affiliations:** Musashino University, Nishitokyo, Tokyo, Japan

## Abstract

“IBASHO” in Japan means a place where people can feel safe, have roles, and recognize their lives as meaningful. If IBASHO helps older adults to see their lives as meaningful, implementing “IBASHO” in the community would possibly contribute to community members’ objective well-being. This research examines the relationship between the activities that make up “IBASHO” and the objective well-being of older adults. Data were gathered by surveying a marginalized community every two years from 2009 to 2019 (Nf882). Multiple regression analysis was conducted. A six-point scale derived from a survey question measured the objective well-being of older adults as the dependent variable. For measuring “IBASHO,” five items asked about engagement in activities such as “hosting a tea meeting” or “holding a hobby workshop,” with the extent of engagement measured using a five-point scale. The total of these five items’ points (Cronbach’s alpha=.860) was analyzed as an independent variable. Gender, age, economic status, health status, and living alone were also included in the analysis. Results show “IBASHO” activity is a significant predictor of the objective well-being of older adults (p < .000) in the full model. Age, economic status, and health status are also significantly associated with well-being. Findings suggest that implementing “IBASHO” within community settings is associated with higher levels of well-being among older adults in the community. “IBASHO” is crucial to realizing the “no one left behind” policy in Japan.

